# Importance of household-level risk factors in explaining micro-epidemiology of asymptomatic malaria infections in Ratanakiri Province, Cambodia

**DOI:** 10.1038/s41598-018-30193-3

**Published:** 2018-08-03

**Authors:** Melanie Bannister-Tyrrell, Set Srun, Vincent Sluydts, Charlotte Gryseels, Vanna Mean, Saorin Kim, Mao Sokny, Koen Peeters Grietens, Marc Coosemans, Didier Menard, Sochantha Tho, Wim Van Bortel, Lies Durnez

**Affiliations:** 1 Institute of Tropical Medicine, Nationalestraat 155, Antwerp, Belgium; 2 National Centre for Parasitology, Entomology and Malaria Control, Phnom Penh, Cambodia; 3 University of Antwerp, Antwerpm, Belgium; 4 Ratanakiri Provincial Health Department, Banlung, Cambodia; 5 Institut Pasteur du Cambodge, Phnom Penh, Cambodia

## Abstract

Heterogeneity in malaria risk is considered a challenge for malaria elimination. A cross-sectional study was conducted to describe and explain micro-epidemiological variation in *Plasmodium* infection prevalence at household and village level in three villages in Ratanakiri Province, Cambodia. A two-level logistic regression model with a random intercept fitted for each household was used to model the odds of *Plasmodium* infection, with sequential adjustment for individual-level then household-level risk factors. Individual-level risk factors for *Plasmodium* infection included hammock net use and frequency of evening outdoor farm gatherings in adults, and older age in children. Household-level risk factors included house wall material, crop types, and satellite dish and farm machine ownership. Individual-level risk factors did not explain differences in odds of *Plasmodium* infection between households or between villages. In contrast, once household-level risk factors were taken into account, there was no significant difference in odds of *Plasmodium* infection between households and between villages. This study shows the importance of ongoing indoor and peridomestic transmission in a region where forest workers and mobile populations have previously been the focus of attention. Interventions targeting malaria risk at household level should be further explored.

## Introduction

Malaria, an infection caused by *Plasmodium* parasites and transmitted by female *Anopheles* mosquitoes, occurs throughout much of the world’s tropical and subtropical regions. Morbidity and mortality due to malaria has decreased worldwide with substantial upscaling of malaria control interventions, and a global malaria eradication goal has been declared^1,2^. However, at regional and local scales, there is substantial variation in distribution of malaria risk, in terms of population at-risk and intensity of transmission, due to climatic, geographic and temporal variations, and variations in human, vector and parasite ecology^3,4^. As malaria control moves towards elimination, there has been increased attention towards estimating and characterizing the total human *Plasmodium* infection prevalence, including asymptomatic and sub-patent infections as well as clinical cases^5^. In most settings, prevalence of asymptomatic infections is substantially higher than clinical malaria cases^6^. Though it remains unclear the extent to which asymptomatic infections contribute to malaria transmission^7^, achieving malaria elimination will likely require reduction of the prevalence of *Plasmodium* infection to zero for sustained interruption of transmission^5^. Characterising and addressing determinants of heterogeneity in total malaria risk, including symptomatic and asymptomatic infections, presents one of the key challenges in malaria elimination^5,8^.

Heterogeneity in malaria risk is frequently conceptualised in terms of ‘hot pops’ and ‘hotspots’. Hot pops refers to population-subgroups defined by a cluster of demographic characteristics who are at higher risk of *Plasmodium* infection^9^. Malaria can also cluster spatially in ‘hotspots’ of higher-than-expected malaria prevalence compared to surrounding areas^10,11^, and can be detected at spatial scales from sub-village to regional-level^12^. The importance of hot pops and hotspots relates to the hypothesis that a ‘parasite reservoir’ of asymptomatic, subpatent infections sustains malaria transmission^13,14^. The assumption behind identifying and targeting malaria hotspots is that infections within hotspots contribute to maintaining the parasite reservoir in a way that infections outside the hotspots do not, and that eliminating malaria from hotspots would lead to interruption of transmission in adjoining areas^11^. Evidence for this premise remains scant; the first trial of its kind showed no effectiveness of hotspot targeting on community-wide malaria prevalence in Kenya^15^.

The Greater Mekong Sub region (GMS) is a global priority region for malaria elimination, due to the multiple emergence and spread of *P*. *falciparum* strains that are resistant to artemisinin-based combination therapies, the current first-line treatments for *P*. *falciparum* worldwide^16^. There is substantial heterogeneity in malaria risk throughout the GMS^17^. Part of this complexity relates to biology and ecology of the parasite and the vector; there are five *Plasmodium* species that cause human malaria infections in the GMS, and there is enormous diversity in the habitats, behaviours and vector competence of the numerous *Anopheles* vectors^17–20^. In the GMS, there is emphasis on ‘mobile and migrant populations’ as a key malaria risk group (i.e. hot pop)^21–25^, and malaria hotspots have been identified at different spatial scales^26–28^. Malaria persists at highest incidence in the GMS in remote forested regions inhabited by ethnic minority populations^17^, who frequently move between houses in the village and at forest farms according to work requirements^22,23,29^. Sleeping overnight at farms was previously shown to increase risk of *Plasmodium* infection^27^, and outdoor evening activities have been postulated as increasing risk of infection through exposure to early evening, outdoor biting vectors^20,29^.

In contrast to the emphasis on identifying hotpops and hotspots, there has been less attention towards explaining whether individual-level, household-level, or higher-level effects are the most important determinants of malaria risk, in the GMS and elsewhere^30,31^. Risk factors for individual-level infection such as age do not explain spatial clustering of malaria except where these demographic characteristics are over-represented, for example at mine sites and in forestry work^31^. Apparent spatial clustering of malaria may represent clustering of malaria at household level, and/or higher-level landscape effects such as local topography or elevation^30,32,33^, or village-level effects such as population size or access to malaria control programs^31^. Household-level determinants of malaria risk are not well characterised in the GMS, in part because it is frequently assumed that most or all malaria transmission occurs away from villages in most settings^21^. However, there are several regions in the GMS where village-based transmission likely persists, and there is some evidence for the importance of household-level characteristics as contributing to malaria risk through mechanisms other than individual-level mobility to higher-risk landscapes. Evidence of local transmission of *P*. *vivax* was found in a settlement on the Thai-Myanmar border that is a focal point for migrant movements, and variation in prevalence of *P*. *vivax* infection was attributed to differences in living conditions rather than migration patterns^34^. In Malaysia, a mix of individual and household-level characteristics relating to the peridomestic environment were risk factors for *P*. *knowlesi* infection^35^.

A recent dry-season survey of malaria prevalence in three villages in malaria hotspots in Ratanakiri province in Eastern Cambodia found several hotspots at sub-village level, however the detected hotspots contained only 60% of the *Plasmodium* infections^26^. A follow-up study did not find evidence for *Plasmodium* hotspots in the same villages in the subsequent year, which will be presented in a forthcoming paper. To better understand the most appropriate units of analysis at which to characterise heterogeneity in malaria risk in this setting, we investigated the relative contribution of individual, household and village-level risk factors for *Plasmodium* infection.

## Results

### Participants

There were 713 respondents to the adolescent and adult survey, and 258 households (including 435 children) completed the household survey, representing 64% of individuals aged 12 years and over, 73% of children and 75% of households in the three villages. Participation in the surveys varied between the three villages (Table 1). In the adolescent and adult survey, older individuals and residents who mainly lived in the village were over-represented, but there were no significant differences in demographic characteristics between surveyed and non-surveyed children (Table 1). Smaller households were somewhat underrepresented in the survey (Table 1). *Plasmodium* infection data was available for 631 (88%) adolescents and adults, and 404 (93%) children. *Plasmodium* infection prevalence by PCR in adolescents and adults in 2016 and/or 2017 was 6.5% in Chamkar San, 10.9% in Phi and 16.6% in Tun, and in children, 4.1% in Chamkar San, 11.9% in Phi and 9.0% in Tun, and declined overall in the three villages between 2016 and 2017 (Table 2). Across both malariometric surveys, all individuals with fever, except two, were negative for *Plasmodium* by PCR. Of the two individuals with fever and positive by PCR, one was positive by rapid diagnostic test (RDT) and was treated with dihydroartemisinin plus piperaquine combination according to the national treatment guidelines, and one had a negative RDT.

**Table 1:** Participation in individual and household surveys.

Characteristic*	Adolescents and adults			Children			Household		
	In survey	Not in survey	p-value	In survey	Not in survey	p-value	In survey	Not in survey	p-value
Village			<0.0001			<0.0001			0.007
Chamkar San	36.02%	32.23%		41.54%	25.00%		36.43%	29.41%	
Phi	31.52%	48.83%		36.82%	50.48%		30.62%	48.04%	
Tun	32.45%	18.95%		21.64%	24.52%		32.95%	22.55%	
Age							N/A		
Median	28 years	24 years	<0.0001	5 years	5 years	0.49			
Sex			0.051			0.92			
Male	48.14%	53.94%		50.87%	50.49%				
Female	51.86%	46.10%		49.13%	49.51%				
Main residence						0.37			
Village	61.79%	46.51%	<0.0001	57.25%	53.43%				
Farm	38.20%	53.49%		42.75%	46.57%				
Household size	N/A			N/A					0.03
0 to 3							27.80%	35.53%	
4 to 5							29.46%	39.47%	
6 to 7							24.07%	9.21%	
8 to 9							10.79%	11.84%	
10 or more							7.88%	3.95%	

*Based on variables collected in 2016 census.

p-values calculated chi-squared tests, except for age, for which Mann Whitney test for differences in distributions was used.

**Table 2:** Prevalence of *Plasmodium* infection by village for respondents to cross-sectional survey.

Village	Population	n *Plasmodium* infections (%) by PCR		
		2016	2017	2016 and/or 2017
Chamkar San	Adolescents/adults	12 (5.6%)	8 (4.5%)	15 (6.5%)
	Children	6 (3.7%)	2 (1.4%)	7 (4.1%)
Phi	Adolescents/adults	19 (11.4%)	2 (2.4%)	20 (10.9%)
	Children	16 (11.7%)	3 (3.9%)	17 (11.9%)
Tun	Adolescents/adults	28 (13.5%)	13 (6.7%)	36 (16.6%)
	Children	6 (7.1%)	4 (5.1%)	8 (9.0%)

### Description of study population

The individual-level characteristics of the study population aged 12 years and over are described in Table 3. Over 90% of the population were of ethnic minorities, and born in their village or district (76%). Most were farmers (85%) or school students (11%). Most (78%) reported sleeping at least sometimes in the village, with 22% only residing at their farm house. Conversely, 26% reported to only reside in their village. The frequency of evening outdoor activities was similar at village and farm locations, with 20% and 22% participating in daily evening gatherings or parties, and 8% and 7% watching TV/DVD daily outdoors in the evening, respectively. Usual bed net use was similar across different locations, with 50% and 49% usually using an insecticide treated net (ITN) or long-lasting insecticidal net (LLIN) at village and farm locations respectively, and 60% reported sleeping under an ITN/LLIN the night before the survey. Similar proportions reported sleeping under untreated market nets (24% in village, 20% at farms), and a few reported sleeping under hammock nets (n = 10 at farms, 5 at village), while small proportions reported not sleeping under any bed net (3% at village, 4% at farms, and 5% the night before the survey). There were 221 (35%) individuals who reported spending time in the forest in the evenings in the past year, mostly for hunting (21%), foraging (22%), fishing (14%) and logging (11%).

**Table 3:** Individual-level characteristics of 713 adolescent and adult individuals participating in the cross-sectional survey.

Demographic		Village activities		Farm activities		Forest activities		Malaria history and prevention	
Variable	n (%)	Variable	n (%)	Variable	n (%)	Variable	n (%)	Variable	n (%)
**Sex**		**Ever sleeps in village**		**Ever sleeps at farm**		**Spends evenings in forest**		**Ever had malaria**	
Male	344 (48.2)	No	159 (22.3)	No	187 (26.2)	No	459 (64.4)	No	263 (36.9)
Female	369 (51.8)	Yes	554 (77.7)	Yes	526 (73.8)	Yes	254 (35.6)	Yes	450 (63.1)
**Age group**		**Frequency of village sleeping in dry season**		**Frequency of farm sleeping in dry season**		**Hunts in evening**		**Preferred care for fever**	
12–15	86 (12.1)	Every night	230 (32.3)	Every night	174 (24.4)	No	562 (78.8)	VMW	28 (3.9)
16–29	292 (41)	At least weekly	196 (27.5)	At least weekly	195 (27.3)	Yes	151 (21.2)	Health centre	469 (65.8)
30–49	222 (31.1)	Monthly or less	123 (17.3)	Monthly or less	109 (15.3)	**Goes logging in evening**		Private provider	142 (19.9)
50+	113 (15.8)	Never	164 (23)	Never	235 (33.0)	No	633 (88.8)	Grocery store	16 (2.2)
**Village**		**Frequency of village sleeping in rainy season**		**Frequency of farm sleeping in rainy season**		Yes	80 (11.2)	Referral hospital	36 (5.0)
Chamkar San	258 (36.2)	Every night	198 (27.8)	Every night	196 (27.5)	**Forages in evening**		Don’t know	22 (3.1)
Phi	224 (31.4)	At least weekly	168 (23.6)	At least weekly	240 (33.7)	No	553 (77.6)	**Usual bed net use at farm**	
Tun	231 (32.4)	Monthly or less	156 (21.9)	Monthly or less	66 (9.3)	Yes	160 (22.4)	No BN	26 (3.7)
**Ethnicity**		Never	191 (26.8)	Never	211 (29.6)	**Goes fishing in evening**		ITN/LLIN	349 (49)
Ethnic minority	655 (91.9)	**Frequency of evening outdoor TV watching**		Frequency of evening outdoor TV watching		No	617 (86.5)	Market net	140 (19.7)
Khmer	58 (8.1)	Every night	60 (8.4)	Every night	47 (6.6)	Yes	96 (13.5)	Hammock net	10 (1.4)
**Occupation**		At least weekly	134 (18.8)	At least weekly	42 (5.9)	**Gold mining in evening**		Does not sleep at farm	187 (26.3)
Farmer	608 (85.3)	Monthly or less	48 (6.7)	Monthly or less	11 (1.5)	No	704 (98.7)	**Usual bed net use in village**	
Student	77 (10.8)	Never	471 (66.1)	Never	613 (86.0)	Yes	9 (1.3)	No BN	22 (3.1)
Other	28 (3.9)	**Frequency of evening indoor TV watching**		**Frequency of evening indoor TV watching**				ITN/LLIN	354 (49.9)
**Duration of residence**		Every night	95 (13.3)	Every night	104 (14.6)			Market net	170 (23.9)
Born here	538 (75.5)	At least weekly	144 (20.2)	At least weekly	74 (10.4)			Hammock net	5 (0.7)
≥10 years	61 (8.6)	Monthly or less	22 (3.1)	Monthly or less	6 (0.8)			Does not sleep in village	159 (22.4)
<10 years	114 (16)	Never	452 (63.4)	Never	529 (74.2)			**Bed net use last night**	
**Lives away part of year**		**Frequency of evening outdoor gatherings**		**Frequency of evening outdoor gatherings**				No BN	32 (4.5)
Another district	28 (3.9)	Every night	143 (20.1)	Every night	154 (21.6)			Program net	425 (59.6)
Another province	13 (1.8)	At least weekly	221 (31)	At least weekly	154 (21.6)			Market net	249 (34.9)
No	672 (94.2)	Monthly or less	111 (15.6)	Monthly or less	81 (11.4)			Hammock net	7 (1.0)
**Main residence**		Never	238 (33.4)	Never	324 (45.4)				
Village	308 (43.2)	**Usual sleeping time at village**		**Usual sleeping time at farm**					
Farm	405 (56.8)	By 7 pm	146 (20.5)	By 7 pm	208 (29.2)				
		By 8 pm	214 (30.0)	By 8 pm	191 (26.8)				
		After 8 pm	194 (27.2)	After 8 pm	126 (17.7)				
		Does not sleep in village	159 (22.3)	Does not sleep at farm	188 (26.4)				

Of the children under 12 years, 51% of children were male, 95% were of indigenous ethnicity, and almost all used a bed net (60% ITN/LLIN, 38% market net) the night before the survey. Most children played outside every night (76%), and had similar frequencies of sleeping at farm/field and village locations in the rainy and dry season as adults (Table 4).

**Table 4:** Individual-level characteristics of 435 children included in the cross-sectional survey.

Demographic		Mobility patterns		Evening activities	
Variable	n (%)	Variable	n (%)	Variable	n (%)
**Sex**		**Frequency of sleeping in village in dry season**		**Bed net use night before survey**	
Male	222 (51.0)	Every night	107 (25.6)	No BN	7 (1.7)
Female	213 (49.0)	At least weekly	111 (26.6)	Program net	247 (59.8)
**Age group**		Monthly or less	95 (22.7)	Market net	159 (38.5)
0–3	125 (28.7)	Never	105 (25.1)	**Frequency of evening outdoor play**	
4–7	160 (36.8)	**Frequency of sleeping in village in rainy season**		Every night	330 (75.9)
8–11	150 (34.5)	Every night	103 (24.6)	At least weekly	66 (15.2)
**Ethnicity**		At least weekly	86 (20.6)	Monthly or less	39 (9.0)
Ethnic minority	393 (95.2)	Monthly or less	101 (24.2)	**Frequency of evening play indoors**	
Khmer	20 (4.8)	Never	128 (30.6)	Every night	317 (72.9)
**Village**		**Frequency of sleeping at farm in dry season**		At least weekly	63 (14.5)
Chamkar San	179 (41.2)	Every night	107 (25.6)	Monthly or less	55 (12.6)
Phi	158 (36.3)	At least weekly	140 (33.5)	**Usual sleeping time**	
Tun	98 (22.5)	Monthly or less	56 (13.4)	Before 8 pm	233 (53.6)
		Never	115 (27.5)	8 pm or later	202 (46.4)
		**Frequency of sleeping at farm in rainy season**			
		Every night	126 (30.1)		
		At least weekly	153 (36.6)		
		Monthly or less	38 (9.1)		
		Never	101 (24.2)		

Most households comprised up to 7 members (71%) (Table 5). Considerable proportions of households had plant-based or plastic walls (35%), floor (23%) or roofs (15%). Most households relied on shallow wells or small streams for their water supply in the dry season (75%), somewhat less in the rainy season (63%). Several different cash crops were grown, including cashew trees (69%), cassava (49%), soybean (27%) and sesame (10%). Most households (58%) had a member who had stayed overnight for hired work on plantations, but few households had hosted a hired worker (4%) in the past year. Common assets included motorbikes (80%), large batteries (74%), solar panels (46%), DVD/MP3 players (39%) and farming machines (e.g. small tractors) (26%). Generators (13%) and TVs (12%) were less common, and satellite dishes (2%), cars (2%) and buffalo or cows (8%) were rare.

**Table 5:** Household-level characteristics of 258 households included in cross-sectional survey.

Household characteristics		House construction characteristics		Household assets		Household agriculture	
Variable	n (%)	Variable	n (%)	Variable	n (%)	Variable	n (%)
**Village**		**Number of steps from ground to house**		**Total number of village houses**		**Crops grown**	
Chamkar San	94 (36.4)	On the ground	59 (22.9)	0	50 (19.4)	Cassava	122 (48.8)
Phi	79 (30.6)	1–5 steps	109 (42.2)	1	204 (79.1)	Soybean	67 (26.8)
Tun	85 (32.9)	6–9 steps	57 (22.1)	2 to 3	4 (1.6)	Peanut	4 (1.6)
**Household size**		10 or more steps	33 (12.8)	**Total number of farm houses**		Cashew	173 (69.2)
0–3	67 (27.8)	**House main roof material**		0	65 (25.2)	Green bean	5 (2.0)
4–5	71 (29.5)	Plant/plastic	39 (15.1)	1	172 (66.7)	Sesame	26 (10.4)
6–7	58 (24.1)	Tin/Tile	219 (84.9)	2 to 4	21 (8.2)	**Household member stayed overnight at cashew or rubber plantation in past year**	
8–9	26 (10.8)	**House main wall material**		**Household property**			
10 or more	19 (7.9)	Plant/plastic	90 (34.9)	Car	6 (2.3)	No	108 (41.9)
**Socioeconomic status**	Wood/tin	168 (65.1)	Motorbike	55 (21.3)	Yes	150 (58.1)	
First (lowest)	61 (23.6)	**House main floor material**		TV	31 (12.0)	**Household hosted hired workers in past year**	
Second	69 (26.7)	Bamboo/earth	58 (22.5)	DVD/MP3 player	101 (39.1)	No	249 (96.5)
Third	93 (36.0)	Wood/concrete	200 (77.5)	Satellite dish	4 (1.6)	Yes	9 (3.5)
Fouth (highest)	35 (13.6)	**House has open walls**		Generator	33 (12.8)		
**Dry season water source**		No	235 (91.1)	Solar panel	118 (45.7)		
Shallow well or stream	193 (74.8)	Yes	23 (8.9)	Home battery	190 (73.6)		
Other	65 (25.2)			Farm machine	66 (25.6)		
**Rainy season water source**				Buffalo or cow	20 (7.8)		
Shallow well or stream	162 (62.8)						
Other	96 (37.2)						

### Individual and household-level risk factors

#### Adolescents and adults

In unadjusted analyses, age group and bed net use the night before the survey were the only individual-level variables significantly associated with increased odds of *Plasmodium* infection (Table S1). Ethnic Khmers, women, and those who spent less time engaging in outdoor evening activities at the farm, had lower odds of *Plasmodium* infection, though these results did not reach statistical significance. Household variables significantly associated with increased odds of *Plasmodium* infection in unadjusted analysis include plant-based/plastic roof, floor or wall materials, lower household socioeconomic status, and owning a satellite dish (Table S2). Household variables significantly associated with reduced odds of *Plasmodium* infection included motorbike ownership, farm machine ownership, growing cassava or soybean crop (Table S2).

In adjusted analyses, in the model with individual-level exposures only (Model 1, Table 6), adolescent or oldest age group, increasing frequency of evening outdoor farm gatherings and hammock net use were associated with increased odds of *Plasmodium* infection. After including village (Model 1a, Table 6), age group was borderline significant, and village was highly significant (p = 0.004). In the individual and household exposures model (Model 2, Table 6), risk factors included increasing frequency of evening outdoor farm gatherings (OR 2.17, 95% CI 1.10–4.25, p = 0.024), hammock net use (OR 21.6, 95% CI 1.84–253.6, p = 0.038), and satellite dish ownership (OR 9.71, 95% CI 2.14–44.0, p = 0.003), whereas age group was no longer significant (p = 0.11). Having wood or tin walls (OR 0.46, 95% CI 0.25–0.83, p = 0.01), owning a farming machine (OR 0.39, 95% CI 0.18–0.85, p = 0.018), and cassava (OR 0.54, 95% CI 0.30–0.99, p = 0.048) and soybean (OR 0.39, 95% CI 0.15–0.99, p = 0.047) crops were associated with reduced odds of *Plasmodium* infection. Adding village to the final model (Model 2a, Table 6) attenuated the effect sizes and significance for cassava and soybean crops, though village was no longer associated with *Plasmodium* infection (p = 0.60).

**Table 6:** Individual and household-level risk factors for *Plasmodium* infection (adolescents and adults).

Variable	Model 1: Individual-level variables			Model 1a: Individual-level variables + village		
	aOR	[95% CI]	p-value	aOR	[95% CI]	p-value
**Age**			0.027			0.057
12–15	1	[1–1]		1	[1–1]	
16–29	0.48	[0.19–1.20]		0.53	[0.22–1.29]	
30–49	0.61	[0.24–1.54]		0.67	[0.28–1.65]	
50+	1.48	[0.55–3.95]		1.44	[0.56–3.68]	
**Evening farm gathering frequency**	2.23	[1.10–4.52]	0.027	2.35	[1.19–4.65]	0.014
**Bed net use night before survey**			0.052			0.028
No BN	1	[1–1]		1	[1–1]	
ITN/LLIN	0.98	[0.23–4.26]		0.74	[0.18–3.09]	
Market net	0.93	[0.20–4.19]		0.61	[0.14–2.66]	
Hammock net	21	[1.49–295.2]		15.9	[1.27–198.1]	
**Village**	N/A			0.0035		
Chamkar San		1	[1–1]			
Phi		1.6	[0.72–3.55]			
Tun		3.38	[1.63–7.00]			
	**Model 2: Individual- and household-level variables**			**Model 2a: Individual- and household-level variables + village**		
	**aOR**	**[95% CI]**	**p-value**	**aOR**	**[95% CI]**	**p-value**
**Age**			0.11			0.10
12–19	1	[1–1]		1	[1–1]	
16–29	0.52	[0.21–1.27]		0.52	[0.22–1.26]	
30–49	0.6	[0.24–1.47]		0.61	[0.25–1.48]	
50+	1.22	[0.48–3.10]		1.24	[0.49–3.11]	
**Evening farm gathering frequency**	2.17	[1.10–4.25]	0.024	2.23	[1.14–4.36]	0.019
**Bed net use night before survey**			0.038			0.035
No BN	1	[1–1]		1	[1–1]	
ITN/LLIN	1.02	[0.25–4.18]		0.9	[0.22–3.75]	
Market net	1.02	[0.24–4.30]		0.87	[0.20–3.76]	
Hammock net	21.6	[1.84–253.6]		18.9	[1.66–216.0]	
**House main wall material**			0.01			0.014
Plant/plastic	1	[1–1]		1	[1–1]	
Wood/tin	0.46	[0.25–0.83]		0.46	[0.25–0.86]	
**Satellite dish ownership**			0.003			0.004
No	1	[1–1]		1	[1–1]	
Yes	9.71	[2.14–44.0]		8.95	[2.05–39.1]	
**Farm machine ownership**			0.018			0.02
No	1	[1–1]		1	[1–1]	
Yes	0.39	[0.18–0.85]		0.4	[0.18–0.87]	
**Household grows cassava crop**			0.048			0.18
No	1	[1–1]		1	[1–1]	
Yes	0.54	[0.30–0.99]		0.62	[0.30–1.25]	
**Household grows soy bean crop**			0.047			0.36
No	1	[1–1]		1	[1–1]	
Yes	0.39	[0.15–0.99]		0.56	[0.16–1.95]	
**Village**	N/A			0.60		
Chamkar San		1	[1–1]			
Phi		1.35	[0.47–3.88]			
Tun		1.66	[0.61–4.51]			

#### Children

Age was the only individual-level variable associated with *Plasmodium* infection in children in the unadjusted analysis (Table S3). Household variables associated with increased malaria risk in children in unadjusted analyses included household water collection from shallow well or stream, in either rainy or dry season, soy bean crop, and having a household member who stayed overnight at rubber or cashew plantations in the past year (Table S4). In adjusted analyses, in the individual-level model (Model 1, Table 7), older age and decreasing frequency of village sleeping in the dry season were associated with increased odds of *Plasmodium* infection. Village was significant after adjustment for individual variables (Model 1a, Table 7). After including household exposures (Model 2, Table 7), older age (OR 8.2, 95% CI 1.62–41.4 for 8–11 year olds compared to 0–3 year olds) remained associated with increased odds, and households with soy bean crops (OR 0.035, 95% CI 0.0036–0.35) had substantially lower odds, but households with peanut crops had higher odds, though with marginal significance (p = 0.06). Village was no longer significant (p = 0.89, Model 2a, Table 7).

**Table 7:** Individual and household-level risk factors for *Plasmodium* infection (children).

Variable	Model 1: Individual-level variables			Model 1a: Individual-level variables + village		
	aOR	[95% CI]	p-value	aOR	[95% CI]	p-value
**Age (years)**			0.0006			0.0006
0–3	1	[1–1]		1	[1–1]	
4–7	3.93	[0.75–20.6]		3.9	[0.73–20.7]	
8–11	11.7	[2.28–59.6]		11.9	[2.27–61.8]	
**Decreasing frequency of village sleeping in dry season**	1.59	[1.01–2.52]	0.047	1.71	[1.01–2.89]	0.04
**Village**	N/A					0.04
Chamkar San				1	[1–1]	
Phi				3.76	[1.07–13.2]	
Tun				4.52	[1.02–20.0]	
	**Model 2: Individual- and household-level variables**			**Model 2a: Individual- and household-level variables + village**		
	**aOR**	**[95% CI]**	**p-value**	**aOR**	**[95% CI]**	**p-value**
**Age (years)**			0.005			0.005
0–3	1	[1–1]		1	[1–1]	
4–7	3.29	[0.64–17.0]		3.25	[0.62–16.9]	
8–11	8.36	[1.66–42.1]		8.2	[1.62–41.4]	
**Decreasing frequency of village sleeping in dry season**	1.56	[0.98–2.48]	0.06	1.51	[0.89–2.57]	0.13
**Household grows soy bean crop**			0.004			0.006
No	1	[1–1]		1	[1–1]	
Yes	0.035	[0.0036–0.35]	0.03	[0.0019–0.36]		
**Household grows peanut crop**			0.06			0.06
No	1	[1–1]		1	[1–1]	
Yes	12.6	[0.92–174.1]		13.2	[0.91–191.4]	
**Village**	N/A					0.89
Chamkar San				1	[1–1]	
Phi				0.72	[0.17–3.02]	
Tun				0.68	[0.12–3.84]	

### Variation between households explained at each stage of model adjustment

In the null model for adolescents and adults, there was evidence of significant variation between households in odds of *Plasmodium* infection (ICC 0.19, p = 0.046) (Table 8). In the individual-level exposures model, the between-household variance increased 13% relative to the null model (Table 8). After including household-level exposures, the between-household variance reduced by 86% relative to the null model and was no longer significant (ICC 0.04, p = 0.37). Adding village to the individual and household model further reduced the between-household variance by 8% (Table 8). Analogously, the median odds ratio decreased from 1.92 to 1.05 between the null and the final model, confirming that there was no remaining household-level effect after adjusting for significant covariates at household-level (Table 8).

**Table 8:** Between-household variance in odds of Plasmodium infection at each stage of model adjustment.

Model	ICC	[95% CI]	p-value	proportional change in variance*	MOR**
**Adolescents and adults**					
Null (empty)	0.19	[0.05–0.51]	0.046	0	1.92
1: Individual	0.24	[0.08–0.54]	0.024	0.13	2.28
2: Individual + household	0.04	[0.00–0.96]	0.37	−0.86	1.12
2a: Individual + household + village	0.02	[0.00–0.99]	0.45	−0.94	1.05
**Children**					
Null (empty)	0.38	[0.14–0.70]	0.011	0	4.03
1: Individual	0.36	[0.11–0.71]	0.023	−0.25	3.68
2: Individual + household	0.22	[0.03–0.73]	0.12	−0.71	2.26
2a: Individual + household + village	0.22	[0.03–0.73]	0.12	−0.71	2.26

*Correction applied to variance of household random effect in adjusted models to scale to variance of null model.

**Median odds ratio.

There was strong evidence of clustering at household level in the null model for children (ICC = 0.38, p = 0.01), which was not attenuated by adjusting for individual-level variables, but was substantially attenuated (71%) by adjusting for household-level variables, at which point there was no statistical evidence for the household-level effect (p = 0.12). Including village as a fixed effect did not further change the ICC (Table 8). Analogously, the median odds ratio changed from 4.03 in the null model to 2.26 in the final model (Table 8). Thus, the median magnitude of the remaining household random effect, though not statistically significant, is larger than the effect of village and frequency of village sleeping, but smaller than the effects of age or crop type.

### Population-average versus cluster-specific fixed effects

In the final adolescent and adult individual and household model (Model 2a, Table 6), the variance of the household random effect was 0.05, corresponding to a shrinkage factor of 0.99, which implies that the modelled cluster-specific odds ratios for the household-level variables are good approximations to population-average odds ratios. For the final children’s model (Model 2a, Table 7), the variance of the random effect was 0.93, corresponding to a shrinkage factor of 0.87. Thus, the approximate population-average odds ratios for household-level effects in the final model are 0.04 (95% CI 0.004–0.41) for growing soy bean crops, and 9.44 (95% CI 0.92–96.67) for peanut crops.

## Discussion

This study found that differences in *Plasmodium* infection prevalence between households and between three villages in Ratanakiri province could be explained by household-level but not individual-level risk factors. In a previous analysis based on data collected in the same setting, village remained a significant risk factor after adjusting for a limited set of individual-level risk factors^26^, in accordance with the findings from the individual-level only risk factor models in the current study. Though the study was restricted to three villages, the three villages were amongst the highest endemic villages in Ratanakiri at the time of selection, and represented distinct transmission and livelihood contexts.

Household level risk-factors explained 86% of between-household variance in *Plasmodium* infection in adolescents and adults, and 71% of between-household variance for children. Higher quality house wall construction materials reduced *Plasmodium* infection risk for adolescents and adults. Though this region is known for vectors that bite in the early evening and outdoors^20^, the finding that house wall material is a risk factor suggests that indoor biting may remain an important component of malaria transmission. The lack of effect of house construction materials for children may suggest differences in actual bed net use or other protective effects that were not captured in this survey. The similar prevalence of *Plasmodium* infection for users of LLINs compared to untreated market nets is likely because the last bed net distribution occurred in 2012 thus by the time the study was conducted, the insecticidal and repellency properties of the distributed LLINS had subsided, and many were observed to be no longer fully intact.

Other household-level risk factors indicate the importance of peridomestic exposure to vector bites around houses and at farms. Satellite dish ownership is likely a proxy for hosting evening gatherings, which can include TV watching, and may indicate that groups of people congregate at these houses, potentially contributing to a pocket of transmission. Though satellite dish ownership was uncommon, the observation that the two *Plasmodium*-positive satellite-dish owning households were located in two different villages suggests that this finding is not confounded by a local feature particular to one village only. This effect persisted after adjustment for socio-economic status, which though non-significant, was in the opposite direction (i.e. protective effect of higher socio-economic status). Farm machine ownership was protective for adolescents and adults but not children, possibly because farmers who have access to farm machines such as tractors spend fewer evening hours tending to their farms. There was negligible change from the unadjusted to the adjusted analyses, including after adjusting for village, which suggests that farm machine ownership is not a proxy for socioeconomic status, frequency of sleeping overnight at farms, or village-specific characteristics.

Crop type also had a significant effect on *Plasmodium* infection prevalence. Soy bean farming remained strongly protective for children even after adjusting for village, however peanut farming, which occurred in Chamkar San and Tun but not Phi (which had the highest *Plasmodium* infection prevalence in children), increased risk. After including village in final model for adolescents and adults, the protective effect of cassava and soybean farming decreased, likely due to collinearity with village. Cassava farming was least common in Tun, which had the highest adolescent and adult *Plasmodium* infection prevalence, and soybean farming occurred mostly in Chamkar San, which had the lowest prevalence. Thus, these associations with household crop types could be causal, which could be explained by the effect of these crops on suitability of local farm landscapes for vector breeding, but crop type may also be a proxy for unmeasured characteristics.

Individual-level risk factors did not explain differences in *Plasmodium* infection prevalence between households or between villages. Apart from age in children, no demographic characteristics were significant individual-level risk factors. Increasing prevalence of *Plasmodium* infection amongst older children likely reflects the age-shift in mean age at first infection in low transmission settings^36^, as well as plausibly increased exposure to biting vectors amongst older children.

Amongst adolescents and adults, evening farm gathering frequency and hammock net use were the significant individual-level variables, which increased the between-household variance. Evening farm gatherings involve groups of people coming together to sit in the environs of the farm house (including the area beneath houses constructed on stilts, inside partially or fully open-walled farm houses, and outside the house) to partake in social drinking (‘parties’) and various ceremonies, mostly without wearing long clothing, and sometimes until late in the evening; thus participating individuals may be highly exposed to early evening, outdoor biting vectors^29,37^. That these groups were observed to often comprise individuals from multiple households may explain why individual-level risk factors increased the between-household variance. There may be a role for spatial repellents to protect people in peri-domestic farm spaces during vector biting hours.

Increased risk of malaria amongst adolescent and adult hammock net users is concerning, because hammocks have been distributed for use in this setting specifically to prevent forest malaria^38^. Though the numbers using hammock nets were small, at least 50% of respondents who slept in a hammock net the night before the survey, or usually used hammock nets at farms and/or in the village, were malaria positive. Though the distributed hammock nets were insecticide-treated and provided by the national malaria control program, the last distribution occurred in 2012, therefore any insecticidal effect had likely subsided. In an elimination context, it is vital to ensure that distributed interventions reduce malaria transmission, and effects of hammock nets under conditions of actual use should be evaluated in a future study.

There were several strengths of this study, including the collection of data on an extensive list of individual and household-level variables, including several for which previous studies in the region have suggested could be linked to increased risk of malaria but for which epidemiological evidence was not available^22,23,29^. The data collection focused on specific activities, behaviours and characteristics that likely directly mediate exposure to vectors, rather than only general risk factors (e.g. sex, ethnicity) that do not indicate potentially causal effects^31^. The multilevel modelling approach used in this study addressed a number of limitations of previous studies. Two multilevel studies of malaria epidemiology in Madagascar^39^ and Ethiopia^40^ included village-level but not household-level random effects, and concluded that village-level effects were important. However, the present study shows the importance of household-level effects in explaining differences between villages. Another study in Ethiopia investigated individual and household effects, but because the study was conducted within one administrative area, the authors could not address whether household-level effects explained differences between villages or analogous higher-level units^41^. A recent study in Peru^42^ compared risk factors in community-specific models to combined models, but reported variances of the random household-level effects were too small to allow for a sequential adjustment strategy as used here. None of these studies corrected for rescaling of variance after inclusion of covariates, which may have biased comparisons of variance between unadjusted and adjusted models. In general, multilevel modelling is underutilized in malaria epidemiology studies, with many studies fitting cluster-level risk factors in single-level models, despite recognition of the importance of contextual-level effects^39^.

Limitations of the study include incomplete sampling, which was mostly related to inaccessibility of households and/or individuals, with frequent heavy rainfalls flooding rough access tracks to outlying households. This is reflected in the under-representation of individuals residing mainly at their farm houses in the sample, especially in Phi village (Table 1). Younger adults, particularly men, were also under-represented in the adolescent and adult survey, though as neither age or sex were independently associated with *Plasmodium* infection in adjusted analyses, this may not have produced substantial bias in the findings. Similarly, though smaller households were under-represented, household size was not associated with odds of *Plasmodium* infection in the adjusted models for adolescents and adults, or children. Apart from village of residence, surveyed children did not significantly differ from non-surveyed children on demographic characteristics, so the findings from analysis of the children’s survey are likely to be least prone to bias. We were only able to conduct this survey at the mid-point between two malariometric surveys, however, as we focused on relatively stable risk factors, and as asymptomatic infections detected in the malariometric surveys were not followed up or treated, infection status in the first malariometric survey was unlikely to induce change in exposure patterns. We had no information on timing of infection, nor could we separate new from recrudescent *P*. *vivax* infections. In this study, species-specific analyses did not allow consistent estimation of whether individual and household-level risk factors explain differences in *Plasmodium* prevalence between the three villages because of correlation between *Plasmodium* species and village. For example, though Tun had the highest prevalence of *Plasmodium* infections overall, no *P*. *malariae* was detected in either year, and *P*. *falciparum* was detected only in 2016. Finally, as this study was only conducted in three villages, it remains unknown whether household-level risk factors would explain differences in *Plasmodium* infection prevalence in a larger number of villages and/or over larger areas. Future studies should include sufficient villages to allow a village-level random effect to be fitted in addition to the household-level random effect. The inclusion of village as a fixed effect rather than a random effect would have biased the estimates of village towards being more strongly associated with the outcome in this study, so the finding that village was no longer significant after adjusting for household risk factors is robust to this bias.

It remains an important area of research to identify strategies to achieve malaria elimination, and whether the individual, household, hotspot or community, in village or non-village locations, should be the primary unit(s) for intervention. Household characteristics have been overlooked as determinants of malaria risk in regions of the GMS where local transmission occurs, with much more emphasis on individual-level characteristics, especially mobility patterns and occupational profiles linked to forest work^21^. This study found no association between *Plasmodium* infection and frequency of village or farm sleeping, in dry or rainy seasons, nor working in the forest, after adjustment for household-level risk factors. There has been insufficient recognition that risk of *Plasmodium* infection in populations with multiple residence patterns may be determined by the effect of characteristics and activities conducted within peridomestic spaces in combination with extent of local vector-supporting landscapes, rather than mobility patterns *per se*^29^. The potential continuing role of indoor transmission argues against any scaling back of the bed net distribution program. Furthermore, rigorous analysis of how risk factors for infection act at different levels of analysis is essential for the meaningful interpretation of spatial clustering of *Plasmodium* infections, whether detected as “hotspots” or using other spatial approaches. At small spatial scales, apparent “hotspots” may reflect aggregated household-level effects, thus characterizing household-level effects should be an integral component of spatial epidemiological approaches.

There may be grounds for considering reactive household-based screening and treatment strategies in this and similar settings. Treating entire households for each confirmed case (with or without screening of household members) could be an effective intervention that could be implemented as part of routine malaria control, especially if more sensitive diagnostic tools for asymptomatic infections become available for operational use^6^. A study of reactive household-based screening and treatment for asymptomatic infections in Western Cambodia showed no effectiveness compared to random screening^43^, but this was attributed to most infections occurring away from the villages in this setting. Further, this study did not analyse the degree of household clustering of *Plasmodium* infection prior to initiation of the intervention. Reactive screening and treatment would likely be less resource intensive than mass drug administration campaigns targeting entire villages or districts, for which emerging results have shown mixed effectiveness in other GMS settings^44^.

## Conclusions

This study showed the importance of household-level risk factors but not individual-level risk factors in explaining differences in *Plasmodium* infection prevalence between households and between villages, and points to the importance of ongoing indoor and outdoor peridomestic transmission in a region where individual mobility patterns have previously been the focus of attention. LLIN coverage should be improved, and interventions targeting malaria risk at household level should be further explored. Amidst the urgency of eliminating malaria in Cambodia and the rest of the GMS, investigating how determinants of *Plasmodium* infections act at different levels should not be overlooked, which is important for understanding local malaria epidemiology and for identifying the most appropriate intervention targets.

## Methods

### Study design and setting

Individual and household-level risk factors for *Plasmodium* infection were investigated using a cross-sectional survey, conducted as part of a two-year study in three villages (Chamkar San, Tun and Phi) in Ratanakiri Province, eastern Cambodia^26^. As part of the two-year study, two dry-season malariometric surveys were carried out in January/February 2016 and March/April 2017, in which fingerprick blood samples were collected for detection of *Plasmodium* infection by nested real-time polymerase chain reaction (PCR), as previously described^26^. During the malariometric surveys, axillary temperature was also measured, and participants were asked whether they had fever in the past 48 h. Individuals with fever or other malaria related symptoms were offered the possibility to take a rapid diagnostic test [CareStart™ Malaria, pLDH/HRP2 COMBO (PAN/Pf)] and if positive, treated according to the national treatment guidelines. The questionnaires in the current study included items on demographic, social, behavioural and environmental characteristics that were putative risk factors for *Plasmodium* infection, based on a framework for micro-epidemiological malaria studies^31^, an exploratory field visit in the three study villages, and previous anthropological and epidemiological studies in the region^22,23,27,29^. The survey was conducted mid-way between the two malariometric surveys, such that the questionnaires included questions about activities during the previous dry season (encompassing the 2016 malariometric survey), and the rainy season (the period leading up to the 2017 malariometric survey).

**Figure 1:**
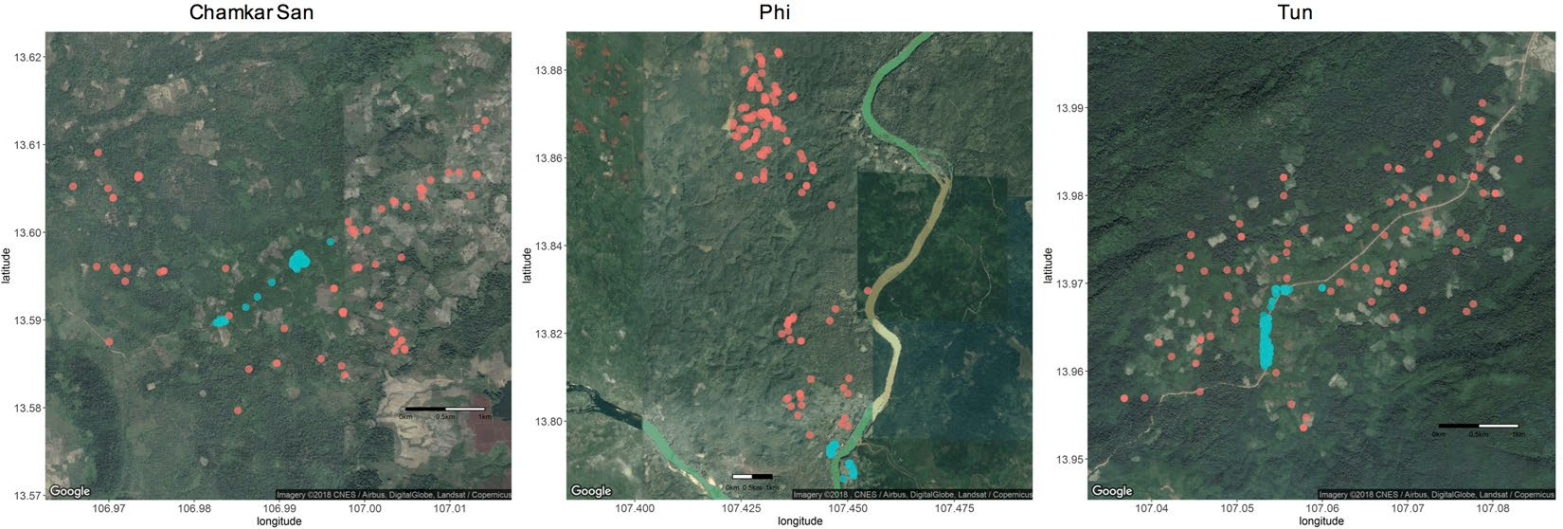
Location of village and farm houses in Chamkar San, Tun and Phi. Red circles indicate farm houses, blue circles indicate village houses. Map Data ©2018, CNES/Airbus, DigitalGlobe, Landsat/Copernicus.

The three study villages represent distinct transmission contexts in Ratanakiri province. Chamkar San is closest to the provincial town of Banlung, and is also the furthest from the forest fringe, with deforestation partly a consequence of increased agriculture and plantations in the area (Fig. 1). Tun and Phi villages are located in forested areas (Fig. 1). Tun comprises a village constructed along a main road, with cashew plantations immediately adjacent to village houses and outlying farms several kilometres from the village. Phi village is close to the Vietnam border, and comprises a village split across the Sesan River, as well as a large cluster of farm houses in a forested area approximately 10 km from the village, and numerous additional outlying farms and fields.

### Participants and sampling

A census conducted in the three villages in January 2016 (n = 1792) formed the basis for the sampling frame^26^, and was updated during the current study as required. Each household and each individual was assigned a unique study identifier. A household was defined as a family unit who habitually reside together in one or more dwellings, including village and farm houses. At any given time, household members may stay in their different houses, and move between residences according to seasonal work requirements.

All resident individuals aged 12 years and older were eligible to participate in the individual survey, and information on children aged 11 years and younger was collected as part of the household survey. The 12 years age cut-off was chosen because adolescents often have different sleeping and activity patterns from their parents^23^. Household heads or their spouses were asked to respond to a household questionnaire, which included questions about the individual-level characteristics of each child residing in the household. Temporary visitors (duration of stay <1 month) were excluded, though few were present.

### Data collection and management

Between September and December 2016, trained surveyors went house by house supported by village guides to administer the individual and household questionnaires. Surveyors rotated between study villages weekly to reduce risk of bias in questionnaire responses. Surveyors made three attempts to reach households and individuals for participation. Survey data was collected on paper forms, and then double-entered and cleaned in EpiData (EpiData Association 2000–2018, Denmark).

### Variables

#### Outcome

The primary outcome was *Plasmodium* infection (all *Plasmodium* species) detected by nested real-time PCR in either or both of the two malariometric surveys. Blood samples were collected and processed as previously described^26^.

#### Exposure variables

The individual questionnaire focused on activity and mobility patterns that may affect exposure to biting vectors in the evening and at night. Frequency of overnight stays at farm and village locations were asked separately by season (rainy/dry). Outdoor evening activities included outdoor TV/DVD watching, indoor TV/DVD watching that occurred outside of a bed net, and evening outdoor gatherings/drinking parties. Frequency of evening activities and usual bed net use were asked separately for farm and village locations. Frequencies were collected as categorical responses (for example ‘every day’, ‘once or twice a week’). Usual sleeping time at farm and village locations were also collected. Usual frequency of evening activities in the forest was difficult for respondents to estimate during piloting, therefore respondents were asked whether they had been in the forest after 5 pm in the past year, and for which activities. Bed net use the night before respondent was interviewed was also collected.

Other individual-level variables included age, sex, ethnicity, occupation, duration of residence and history of malaria. Age was initially examined in 5-year age groups, and then revised to four age groupings of 12–15 years, 16–29, 30–49, and 50 years or older, with *Plasmodium* infection prevalence approximately homogenous at different ages within each age band. Age was not used as a continuous variable because there were imbalanced proportions of respondents reporting their age as 20, 30, 40 or 50 years, reflecting uncertainty about precise age amongst this population. Ethnicity was categorized as ‘ethnic minority’ or ‘Khmer’, as specific ethnicity co-varied too substantially with village to allow independent estimation.

A reduced set of individual-level variables were collected for children 11 years or under, including age (grouped as 0–3, 4–7 and 8–11 years), sex, ethnicity, frequency of overnight stays at farm and village locations by season, frequency of outdoor evening play, usual bed time and bed net use the night before the survey.

**Figure 2:**
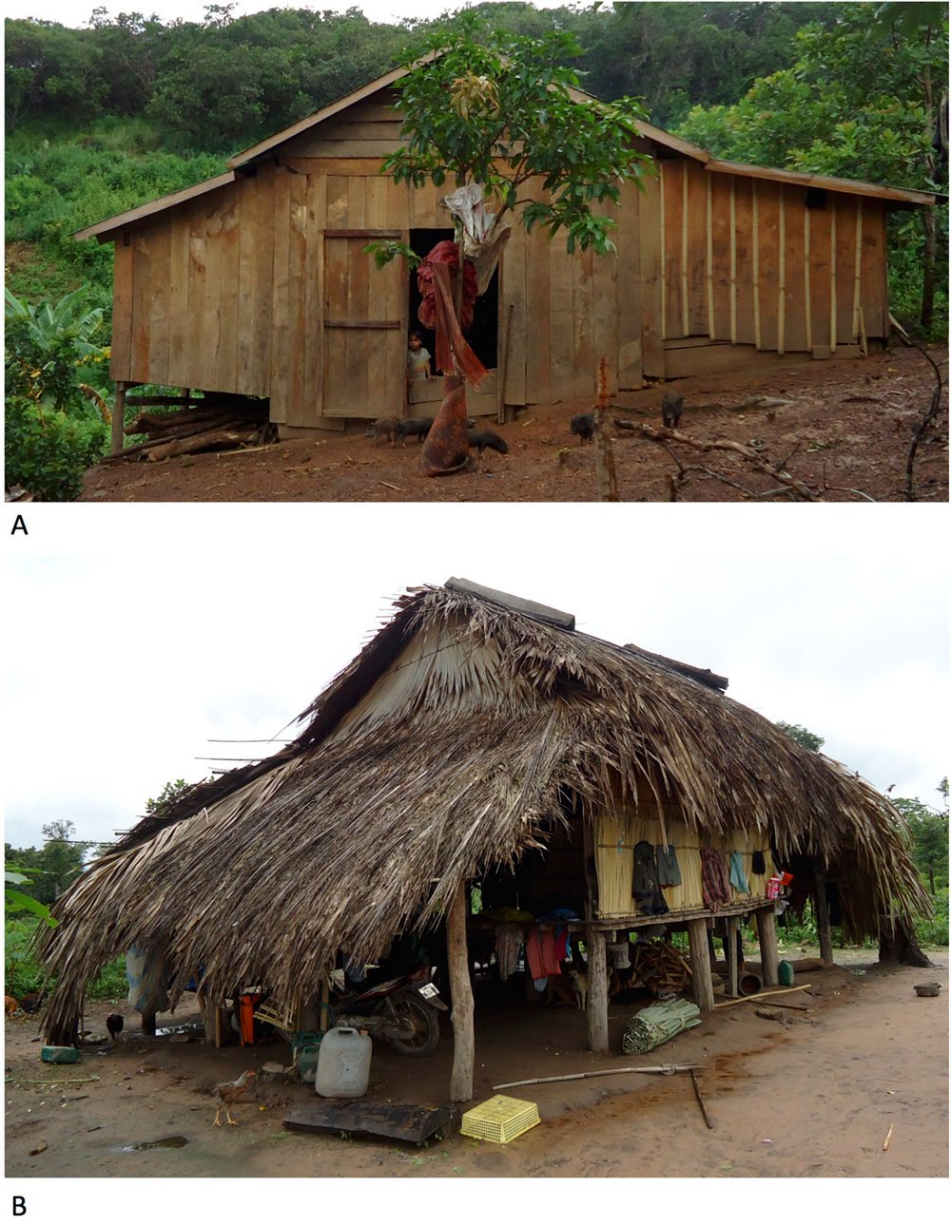
Example of different housing styles in the study villages. Panel A: Ground-level house constructed of wood walls and floor, and tin roof. Panel B: Elevated house constructed of bamboo walls and floor, and plant-based (palm frond) roof. Photographs taken by author Set Srun.

Household-level exposure variables included household size, house construction, total number of houses owned, farms and fields owned, household wealth, cash crops grown, household water source and household mobility. Having a household member who stayed overnight at a plantation in the past year, and households hosting hired workers overnight, were also recorded. A household wealth index was constructed using principal components analysis on 16 variables reflecting household assets (car, motorbike, bicycle, TV, DVD/MP3 player, generator, solar panel, home battery, farm machine (e.g. small tractors), buffalo or cow ownership, house construction (floor, wall and roof materials categorized as wood/tin/concrete versus plant-based [bamboo/thatch/palm leaves] or plastic) and number of farms and fields owned. The first principal component (20.4% explained variance), onto which motorbike, solar panel, battery and DVD/MP3 player ownership as well as roof and wall material loaded most strongly (eigenvectors >0.3), was retained as a household wealth index score. K-means clustering analysis was used to partition the wealth index score into 4 groups. Partition methods (specifying cut points versus cluster analysis) and number of groups (3, 4 or 5) were compared and the final model selected so that differences in mean scores between adjacent categories were similar^45^. House construction variables were recorded for the house where the interview was conducted, which was the household main residence at the time of the survey. An example of the different housing styles in the study villages is shown in Fig. 2.

### Statistical analyses

Two-level logistic regression models with a random intercept fitted for each household were used to model the odds of individual *Plasmodium* infection. There were two objectives for the multilevel analysis; firstly, to identify risk factors for *Plasmodium* infection at individual and household level; secondly, to assess whether differences in odds of *Plasmodium* infection between households and between villages remained unexplained after adjustment for individual and household risk factors. To achieve these objectives, a step-wise modelling approach was used, as follows. First, a null model with household random intercepts only was fitted to calculate the crude household-level variation in odds of *Plasmodium* infection. Second, crude odds ratios and 95% confidence intervals were calculated for each individual-level exposure variable, and included in a combined multivariable model if p < 0.20. Manual backwards stepwise selection was used to retain variables significant at p < 0.05, with age retained as an *a priori* confounder. Village was then added as a fixed effect to determine if individual-level variables explained variation in odds of *Plasmodium* infection between villages. Household-level variables were similarly selected, and then added to the individual model, and the explained between-household variance was determined. Village was added to the final model as a fixed effect, and its significance assessed.

Challenges interpreting the results of multilevel logistic regression models include the interpretation of cluster-specific odds ratios, and making appropriate interpretations of the variance of the cluster-level effect within and between models. These were addressed as follows:

The covariate odds ratios in multilevel logistic regression models have a cluster-specific interpretation^46^. For individual-level characteristics, the interpretation is that the odds ratios reflect the change in odds of the outcome at each level of the exposure variable for individuals within the same cluster and conditioned on all other covariates. However for cluster-level effects, the interpretation is the change in odds of the outcome at each level of the exposure variable relative to the baseline category within the same cluster (and conditioned on covariates), which is problematic because cluster-level values are constant for all individuals within a cluster^46^. To address the difficulty in interpreting household-specific fixed effects, the approximate marginal (i.e. population average) effects were estimated by calculating and applying a shrinkage factor (equation 2 in^46^) to the household-level log-odds ratios and presented as supplementary to the main results.

The relative importance of individual-level compared to household-level effects in each model was assessed in two ways. First, the intra-class correlation coefficients (ICC) were calculated at each stage of model adjustment. The latent response formulation was used to estimate the between-subject variance on the log-odds scale, which assumes that underlying the observed binary outcome (*Plasmodium* infection) is a latent continuous variable for the propensity to be *Plasmodium* infected, which manifests as a binary variable once an unobserved threshold is reached^46^. This approach allows both the cluster-level and individual-level variance to be measured on the log-odds scale, with the between-individual variance following the logistic distribution and fixed at π^2^/3. Thus, the ICC, which expresses the proportion of total variance that is due to the household-level variance can be estimated. Likelihood ratio tests were used to assess the null hypothesis that the ICC was equal to zero; i.e., no evidence of household-level clustering. To complement the interpretation of the ICC, median odds ratios (MOR) were also calculated^47^. MORs are a function of the household-level variance only, and can be interpreted as the increased median odds of *Plasmodium* infection if an individual living in one household moved to a household with higher odds of *Plasmodium* infection, conditioned on covariates^48^. A MOR of 1 indicates no difference between households. As it is expressed as an odds ratio, the magnitude of the household-level random effect can be directly compared to the magnitude of the individual or household-level risk factor odds ratios (or the inverse of the odds ratio, for protective factors)^48^.

The proportion of explained variance at successive stages of model adjustment was calculated the difference between the variance of the random effect in each adjusted model and the variance of the random effect in the null model, expressed as a proportion of the variance of the random effect in the null model. However, as a consequence of the fixed between-individual variance in the latent response formulation, adjustment for individual-level covariates rescales the cluster-level variance, such that household-level variance may appear to increase^46^. Thus, a variance scaling factor (the total variance of the null model as a proportion of the total variance of the adjusted model) was applied to rescale the random variance of the adjusted models to that of the null model, which permits direct comparison at each stage of model adjustment^46^.

In the analyses, frequency variables were first fitted as categorical variables, and then as frequency-weighted continuous variables for comparison; the latter were preferred as long as effect estimates for covariates did not meaningfully shift (>10%) compared to fitting categorical variables. Total number of farm houses and total number of village houses were fitted as linear variables. Missing data were addressed by complete case analysis. P-values were calculated using likelihood ratio tests. Statistical analyses were conducted in Stata/IC v13.1 (StataCorp, Lakeway Dr., College Station, Texas); specifically, multilevel logistic regression models and ICCs were estimated using *xtlogit*, median odds ratios were calculated using *xtrho*, and marginal odds ratios and variance scaling factors were calculated directly^46^.

### Ethical approval and informed consent

Study protocols were approved by the institutional review boards of the National Ethical Committee on Health Research of Cambodia (Approval 309NECHR), the Institutional Review Board of the Institute of Tropical Medicine Antwerp (Approval IRB/AB/ac/119) and the Ethical Committee of the University of Antwerp (Approval B300201525582). After the study information sheet was read out, or provided to literate individuals, verbal informed consent to participate in the survey was obtained and documented. For minors, the informed consent of their parent or legal guardian was required, in addition to their own assent to participate. All individuals were free to refuse or withdraw participation at any time. We confirm that all research was performed in accordance with the Declaration of Helsinki Ethical Principles for Medical Research involving Human Subjects, and all other relevant guidelines and regulations.

### Data availability

The datasets used and/or analysed during the current study are available from the corresponding author on reasonable request.

## Electronic supplementary material

Supplementary Tables
