# Supplementary material for: Importance of household-level risk factors in explaining micro-epidemiology of asymptomatic malaria infections in Ratanakiri Province, Cambodia
Source: Sci Rep. 2018 Aug 3;8:11643. doi: 10.1038/s41598-018-30193-3 (PMC6076298; doi:10.1038/s41598-018-30193-3)
Supplement: Supplementary file 1 — Supplementary Tables [file 41598_2018_30193_MOESM1_ESM.pdf]

Article Overview

Importance of household-level risk factors in explaining micro-epidemiology of asymptomatic malaria infections in Ratanakiri Province, Cambodia

Melanie Bannister-Tyrrell* (mbannister@itg.be)
Institute of Tropical Medicine Antwerp, Belgium
Set Srun (setsrun@gmail.com)
National Centre for Parasitology, Entomology and Malaria Control, Phnom Penh, Cambodia
Vincent Sluydts (vincent.sluydts@uantwerpen.be)
Institute of Tropical Medicine Antwerp, Belgium and University of Antwerp, Belgium
Charlotte Gryseels (cgryseels@itg.be)
Institute of Tropical Medicine Antwerp, Belgium
Vanna Mean (vannamean@yahoo.com)
Ratanakiri Provincial Health Department, Banlung, Cambodia
Saorin Kim (ksaorin@pasteur-kh.org)
Institut Pasteur du Cambodge, Phnom Penh, Cambodia
Mao Sokny (soknym@yahoo.com)
National Centre for Parasitology, Entomology and Malaria Control, Phnom Penh, Cambodia
Koen Peeters Grietens (kpeeters@itg.be)
Institute of Tropical Medicine Antwerp, Belgium
Marc Coosemans (mcoosemans@itg.be)
Institute of Tropical Medicine Antwerp, Belgium and University of Antwerp, Belgium
Didier Menard (didier.menard@pasteur.fr)
Institut Pasteur du Cambodge, Phnom Penh, Cambodia
Sochantha Tho (thosochantha@gmail.com)
National Centre for Parasitology, Entomology and Malaria Control, Phnom Penh, Cambodia
Wim Van Bortel (wvanbortel@itg.be)
Institute of Tropical Medicine Antwerp, Belgium
Lies Durnez (lies.durnez@uantwerpen.be)
Institute of Tropical Medicine Antwerp, Belgium and University of Antwerp, Belgium

Supplementary file contents

Table S1: Unadjusted odds ratios for association between individual-level variables and odds of *Plasmodium* infection (adolescents and adults)
Table S2: Unadjusted odds ratios for association between household-level variables and odds of *Plasmodium* infection (adolescents and adults)
Table S3: Unadjusted odds ratios for association between individual-level variables and odds of *Plasmodium* infection (children)
Table S4: Unadjusted odds ratios for association between household-level variables and odds of *Plasmodium* infection (children)

Table S1: Unadjusted odds ratios for association between individual-level variables and odds of *Plasmodium* infection (adolescents and adults)

Variable	n (%)			OR [95% CI]	p-value
	Total	*Plasmodium -*	*Plasmodium +*		
		Demographic variables			
**Village**					0.007
Chamkar San	231 (36.6)	216 (38.6)	15 (21.1)	1 [1-1]	
Phi	183 (29.0)	163 (29.1)	20 (28.2)	1.8 [0.85-3.83]	
Tun	217 (34.4)	181 (32.3)	36 (50.7)	2.98 [1.49-5.96]	
**Age group**					0.03
12-15	78 (12.4)	67 (12)	11 (15.5)	1 [1-1]	
16-29	250 (39.6)	229 (40.9)	21 (29.6)	0.48 [0.20-1.15]	
30-49	202 (32)	182 (32.5)	20 (28.2)	0.57 [0.24-1.37]	
50+	101 (16)	82 (14.6)	19 (26.8)	1.28 [0.52-3.19]	
**Sex**					0.14
Male	295 (46.8)	256 (45.7)	39 (54.9)	1 [1-1]	
Female	336 (53.2)	304 (54.3)	32 (45.1)	0.66 [0.39-1.12]	
**Ethnicity**					0.2
Ethnic minority	580 (91.9)	512 (91.4)	68 (95.8)	1 [1-1]	
Khmer	51 (8.1)	48 (8.6)	3 (4.2)	0.47 [0.13-1.68]	
**Occupation**					0.53
Farmer	541 (85.7)	480 (85.7)	61 (85.9)	1 [1-1]	
Student	68 (10.8)	59 (10.5)	9 (12.7)	1.33 [0.58-3.04]	
Other	22 (3.5)	21 (3.8)	1 (1.4)	0.39 [0.047-3.24]	
**Duration of residence**					0.5
Born here	484 (76.7)	426 (76.1)	58 (81.7)	1 [1-1]	
10 or more years	57 (9)	51 (9.1)	6 (8.5)	0.81 [0.31-2.14]	
<10 years	90 (14.3)	83 (14.8)	7 (9.9)	0.59 [0.24-1.44]	
**Lives away part of year**					0.93
Yes- another district in Rattanakiri	23 (3.6)	21 (3.8)	2 (2.8)	1 [1-1]	
Yes- another province	9 (1.4)	8 (1.4)	1 (1.4)	1.84 [0.11-30.3]	
No	599 (94.9)	531 (94.8)	68 (95.8)	1.59 [0.32-7.93]	

**Main residence**						0.19
Village	277 (43.9)	251 (44.8)	26 (36.6)	1	[1-1]	
Farm	354 (56.1)	309 (55.2)	45 (63.4)	1.45	[0.81-2.59]	
		Village related variables				
**Ever sleeps in village**						0.43
No	137 (21.7)	119 (21.2)	18 (25.4)	1	[1-1]	
Yes	494 (78.3)	441 (78.8)	53 (74.6)	0.74	[0.38-1.44]	
**Frequency of village sleeping in dry season**						0.84
Every night	210 (33.3)	189 (33.8)	21 (29.6)	1	[1-1]	
At least weekly	176 (27.9)	157 (28)	19 (26.8)	1.12	[0.54-2.31]	
Monthly or less	104 (16.5)	91 (16.2)	13 (18.3)	1.32	[0.58-3.01]	
Never	141 (22.3)	123 (22)	18 (25.4)	1.44	[0.66-3.14]	
**Frequency of village sleeping in rainy season**						0.23
Every night	178 (28.2)	164 (29.3)	14 (19.7)	1	[1-1]	
At least weekly	149 (23.6)	133 (23.8)	16 (22.5)	1.46	[0.64-3.32]	
Monthly or less	139 (22)	118 (21.1)	21 (29.6)	2.16	[0.97-4.78]	
Never	165 (26.1)	145 (25.9)	20 (28.2)	1.75	[0.78-3.95]	
**Frequency of watching TV outside after 5pm in village**						0.08
Every night	53 (8.4)	49 (8.8)	4 (5.6)	1	[1-1]	
At least weekly	114 (18.1)	96 (17.1)	18 (25.4)	2.38	[0.70-8.09]	
Monthly or less	41 (6.5)	33 (5.9)	8 (11.3)	3.04	[0.75-12.3]	
Never	423 (67)	382 (68.2)	41 (57.7)	1.28	[0.41-4.02]	
**Frequency of TV watching inside house but outside bed net after 5pm in village**						0.77
Every night	87 (13.8)	80 (14.3)	7 (9.9)	1	[1-1]	
At least weekly	132 (20.9)	116 (20.7)	16 (22.5)	1.66	[0.60-4.58]	
Monthly or less	20 (3.2)	18 (3.2)	2 (2.8)	1.31	[0.22-7.92]	
Never	392 (62.1)	346 (61.8)	46 (64.8)	1.64	[0.66-4.11]	
**Frequency of evening gatherings outside after 5pm in village**						0.48
Every night	129 (20.4)	112 (20)	17 (23.9)	1	[1-1]	
At least weekly	195 (30.9)	177 (31.6)	18 (25.4)	0.66	[0.31-1.42]	
Monthly or less	100 (15.8)	91 (16.2)	9 (12.7)	0.63	[0.25-1.58]	
Never	207 (32.8)	180 (32.1)	27 (38)	0.99	[0.48-2.05]	
**Village usual sleeping time**						0.79
By 7pm	126 (20)	114 (20.4)	12 (16.9)	1	[1-1]	
By 8pm	195 (30.9)	172 (30.7)	23 (32.4)	1.37	[0.61-3.08]	
After 8pm	173 (27.4)	155 (27.7)	18 (25.4)	1.13	[0.49-2.63]	
Does not sleep in village	137 (21.7)	119 (21.2)	18 (25.4)	1.6	[0.66-3.89]	

			Farm related variables			
**Ever sleeps at farm or field**						0.18
No	166 (26.3)	152 (27.1)	14 (19.7)	1	[1-1]	
Yes	465 (73.7)	408 (72.9)	57 (80.3)	1.6	[0.81-3.19]	
**Frequency of farm or field sleeping in dry season**						0.78
Every night	151 (23.9)	131 (23.4)	20 (28.2)	1	[1-1]	
At least weekly	169 (26.8)	151 (27)	18 (25.4)	0.68	[0.31-1.50]	
Monthly or less	100 (15.8)	88 (15.7)	12 (16.9)	0.83	[0.34-2.01]	
Never	211 (33.4)	190 (33.9)	21 (29.6)	0.62	[0.29-1.36]	
**Frequency of farm or field sleeping in rainy season**						0.38
Every night	168 (26.6)	147 (26.2)	21 (29.6)	1	[1-1]	
At least weekly	212 (33.6)	184 (32.9)	28 (39.4)	0.96	[0.48-1.93]	
Monthly or less	63 (10)	56 (10)	7 (9.9)	0.84	[0.30-2.32]	
Never	188 (29.8)	173 (30.9)	15 (21.1)	0.54	[0.24-1.21]	
**Frequency of TV watching outside at farm/field after 5pm**						0.9
Every night	42 (6.7)	36 (6.4)	6 (8.5)	1	[1-1]	
At least weekly	38 (6)	33 (5.9)	5 (7)	0.78	[0.19-3.19]	
Monthly or less	9 (1.4)	8 (1.4)	1 (1.4)	0.73	[0.062-8.58]	
Never	542 (85.9)	483 (86.2)	59 (83.1)	0.62	[0.22-1.77]	
**Frequency of TV watching inside house but outside bed net after 5pm at farm/field**						0.63
Every night	91 (14.4)	78 (13.9)	13 (18.3)	1	[1-1]	
At least weekly	65 (10.3)	59 (10.5)	6 (8.5)	0.55	[0.18-1.71]	
Monthly or less	5 (0.8)	5 (0.9)	0 (0)	1	[1-1]	
Never	470 (74.5)	418 (74.6)	52 (73.2)	0.75	[0.35-1.58]	
				626		
**Frequency of evening gatherings outside after 5pm at farm/field**						0.12
Every night	136 (21.6)	113 (20.2)	23 (32.4)	1	[1-1]	
At least weekly	132 (20.9)	119 (21.2)	13 (18.3)	0.47	[0.20-1.08]	
Monthly or less	75 (11.9)	69 (12.3)	6 (8.5)	0.35	[0.12-1.03]	
Never	288 (45.6)	259 (46.2)	29 (40.8)	0.48	[0.24-0.97]	
**Farm/field usual sleeping time**						0.21
By 7pm	180 (28.5)	162 (28.9)	18 (25.4)	1	[1-1]	
By 8pm	169 (26.8)	143 (25.5)	26 (36.6)	1.79	[0.87-3.71]	
After 8pm	115 (18.2)	102 (18.2)	13 (18.3)	1.18	[0.51-2.74]	
Does not sleep at farm	167 (26.5)	153 (27.3)	14 (19.7)	0.8	[0.36-1.81]	

			Forest related variables			
**Spent evening in forest in past year**						0.62
No	410 (65)	362 (64.6)	48 (67.6)	1	[1-1]	
Yes	221 (35)	198 (35.4)	23 (32.4)	0.9	[0.51-1.58]	
**Went hunting in forest after 5pm**						0.29
No	501 (79.4)	448 (80)	53 (74.6)	1	[1-1]	
Yes	130 (20.6)	112 (20)	18 (25.4)	1.4	[0.75-2.61]	
**Went logging in forest after 5pm**						0.83
No	564 (89.4)	500 (89.3)	64 (90.1)	1	[1-1]	
Yes	67 (10.6)	60 (10.7)	7 (9.9)	0.9	[0.37-2.19]	
**Went foraging in forest after 5pm**						0.45
No	493 (78.1)	440 (78.6)	53 (74.6)	1	[1-1]	
Yes	138 (21.9)	120 (21.4)	18 (25.4)	1.32	[0.71-2.47]	
**Went fishing after 5pm**						0.08
No	548 (86.8)	491 (87.7)	57 (80.3)	1	[1-1]	
Yes	83 (13.2)	69 (12.3)	14 (19.7)	1.98	[0.96-4.07]	
**Went gold mining after 5pm**						0.99
No	622 (98.6)	552 (98.6)	70 (98.6)	1	[1-1]	
Yes	9 (1.4)	8 (1.4)	1 (1.4)	1	[0.098-10.3]	
**Other activities in forest after 5pm**						0.82
No	620 (98.3)	550 (98.2)	70 (98.6)	1	[1-1]	
Yes	11 (1.7)	10 (1.8)	1 (1.4)	0.87	[0.094-7.98]	
			Malaria history and treatment seeking behaviour variables			
**Ever had malaria**						0.08
No	229 (36.3)	210 (37.5)	19 (26.8)	1	[1-1]	
Yes	402 (63.7)	350 (62.5)	52 (73.2)	1.69	[0.93-3.05]	
**Preferred first place to visit for fever treatment**						0.96
VMW	28 (4.4)	25 (4.5)	3 (4.2)	1	[1-1]	
Health centre	420 (66.6)	370 (66.1)	50 (70.4)	1.1	[0.29-4.26]	
Private provider	125 (19.8)	113 (20.2)	12 (16.9)	0.85	[0.20-3.70]	
Grocery store	14 (2.2)	12 (2.1)	2 (2.8)	1.34	[0.16-11.2]	
Referral hospital	29 (4.6)	26 (4.6)	3 (4.2)	0.92	[0.14-5.95]	
Don't know	15 (2.4)	14 (2.5)	1 (1.4)	0.54	[0.044-6.70]	

		Malaria prevention related variables			
**Usual bed net use at farm**					0.28
No BN	16 (2.5)	15 (2.7)	1 (1.4)	1 [1-1]	
ITN/LLIN	315 (50)	276 (49.4)	39 (54.9)	2.66 [0.29-24.4]	
Market net	127 (20.2)	112 (20)	15 (21.1)	2.5 [0.26-24.2]	
Hammock net	6 (1)	4 (0.7)	2 (2.8)	13.1 [0.66-260.4]	
Does not sleep at farm	166 (26.3)	152 (27.2)	14 (19.7)	1.61 [0.17-15.5]	
**Usual bed net use in village**					0.06
No BN	16 (2.5)	14 (2.5)	2 (2.9)	1 [1-1]	
ITN/LLIN	318 (50.6)	282 (50.4)	36 (52.2)	0.92 [0.17-4.90]	
Market net	153 (24.4)	142 (25.4)	11 (15.9)	0.53 [0.091-3.09]	
Hammock net	4 (0.6)	2 (0.4)	2 (2.9)	12.4 [0.71-218.0]	
Does not sleep in village	137 (21.8)	119 (21.3)	18 (26.1)	1.18 [0.21-6.68]	
**Bed net use night before survey**					0.005
No BN	24 (3.8)	21 (3.8)	3 (4.2)	1 [1-1]	
ITN/LLIN	381 (60.4)	337 (60.2)	44 (62)	1.03 [0.26-4.08]	
Market net	221 (35)	200 (35.7)	21 (29.6)	0.87 [0.21-3.65]	
Hammock net	5 (0.8)	2 (0.4)	3 (4.2)	18.8 [1.51-235.2]	

Table S2: Unadjusted odds ratios for association between household-level variables and odds of *Plasmodium* infection (adolescents and adults)

Variable	n (%)			OR	[95% CI]	p-value
	Total (%)	*Plasmodium* -	*Plasmodium* +			
			Household characteristics			
**Household size**						0.18
0-3	112 (18.2)	93 (17.1)	19 (27.1)	1	[1-1]	
4-5	175 (28.5)	156 (28.6)	19 (27.1)	0.58	[0.27-1.26]	
6-7	165 (26.8)	145 (26.6)	20 (28.6)	0.62	[0.28-1.37]	
8-9	86 (14)	80 (14.7)	6 (8.6)	0.34	[0.11-1.00]	
10 or more	77 (12.5)	71 (13)	6 (8.6)	0.39	[0.13-1.19]	
**Socioeconomic status**						0.02
First (lowest)	123 (19.9)	99 (18.1)	24 (33.8)	1	[1-1]	
Second	149 (24.1)	132 (24.1)	17 (23.9)	0.51	[0.23-1.11]	
Third	241 (39)	220 (40.2)	21 (29.6)	0.36	[0.17-0.76]	
Fouth (highest)	105 (17)	96 (17.6)	9 (12.7)	0.33	[0.13-0.88]	
			House construction characteristics			
**Number of steps from ground to house**						0.27
On the ground	135 (21.8)	116 (21.2)	19 (26.8)	1	[1-1]	
1-5 steps	249 (40.3)	218 (39.9)	31 (43.7)	0.79	[0.38-1.64]	
6-9 steps	142 (23)	132 (24.1)	10 (14.1)	0.41	[0.16-1.04]	
10 or more steps	92 (14.9)	81 (14.8)	11 (15.5)	0.72	[0.28-1.87]	
**House main roof material**						0.03
Plant/plastic	87 (14.1)	71 (13)	16 (22.5)	1	[1-1]	
Tin/Tile	531 (85.9)	476 (87)	55 (77.5)	0.47	[0.23-1.00]	
**House main wall material**						<0.0001
Plant/plastic	228 (36.9)	188 (34.4)	40 (56.3)	1	[1-1]	
Wood/tin	390 (63.1)	359 (65.6)	31 (43.7)	0.37	[0.21-0.66]	
**House main floor material**						<0.0001
Bamboo/earth	133 (21.5)	106 (19.4)	27 (38)	1	[1-1]	
Wood/concrete	485 (78.5)	441 (80.6)	44 (62)	0.35	[0.18-0.66]	
**House has open walls**						0.1
No	206 (33.3)	179 (32.7)	27 (38)	1	[1-1]	
Partially (gaps in walls)	358 (57.9)	324 (59.2)	34 (47.9)	0.68	[0.37-1.27]	
Yes- one or more walls completely open	54 (8.7)	44 (8)	10 (14.1)	1.62	[0.62-4.19]	

		Household assets				
**Total number of village houses**						0.86
0	124 (20.1)	108 (19.7)	16 (22.5)	1	[1-1]	
1	484 (78.3)	430 (78.6)	54 (76.1)	0.75	[0.36-1.54]	
2 to 3	10 (1.6)	9 (1.6)	1 (1.4)	1.01	[0.076-13.3]	
**Total number of farm houses**						0.06
0	134 (21.7)	127 (23.2)	7 (9.9)	1	[1-1]	
1	418 (67.6)	362 (66.2)	56 (78.9)	2.98	[1.24-7.15]	
2	59 (9.5)	53 (9.7)	6 (8.5)	2.1	[0.59-7.40]	
3	3 (0.5)	2 (0.4)	1 (1.4)	10.8	[0.50-234.3]	
4	4 (0.6)	3 (0.5)	1 (1.4)	6.99	[0.37-133.3]	
**Total number of field houses**						0.1
0	598 (96.8)	527 (96.3)	71 (100)	1	[1-1]	
1	20 (3.2)	20 (3.7)	0 (0)	1	[1-1]	
				598		
**Car ownership**						0.17
No	604 (97.7)	533 (97.4)	71 (100)	1	[1-1]	
Yes	14 (2.3)	14 (2.6)	0 (0)	1	[1-1]	
				604		
**Motorbike ownership**						0.006
No	103 (16.7)	83 (15.2)	20 (28.2)	1	[1-1]	
Yes	515 (83.3)	464 (84.8)	51 (71.8)	0.42	[0.21-0.82]	
**Bicycle ownership**						0.09
No	473 (76.5)	413 (75.5)	60 (84.5)	1	[1-1]	
Yes	145 (23.5)	134 (24.5)	11 (15.5)	0.55	[0.26-1.16]	
**TV ownership**						0.88
No	536 (86.7)	474 (86.7)	62 (87.3)	1	[1-1]	
Yes	82 (13.3)	73 (13.3)	9 (12.7)	0.81	[0.32-2.00]	
**DVD/MP3 player ownership**						0.5
No	351 (56.8)	308 (56.3)	43 (60.6)	1	[1-1]	
Yes	267 (43.2)	239 (43.7)	28 (39.4)	0.81	[0.45-1.46]	
**Satellite dish ownership**						0.03
No	605 (97.9)	538 (98.4)	67 (94.4)	1	[1-1]	
Yes	13 (2.1)	9 (1.6)	4 (5.6)	3.51	[0.76-16.2]	
**Generator ownership**						0.72
No	531 (85.9)	469 (85.7)	62 (87.3)	1	[1-1]	
Yes	87 (14.1)	78 (14.3)	9 (12.7)	0.79	[0.33-1.91]	

**Solar panel ownership**					0.82
No	297 (48.1)	262 (47.9)	35 (49.3)	1 [1-1]	
Yes	321 (51.9)	285 (52.1)	36 (50.7)	0.94 [0.53-1.67]	
**Home battery ownership**					0.21
No	130 (21)	111 (20.3)	19 (26.8)	1 [1-1]	
Yes	488 (79)	436 (79.7)	52 (73.2)	0.69 [0.36-1.33]	
**Farm machine ownership**					0.005
No	434 (70.2)	374 (68.4)	60 (84.5)	1 [1-1]	
Yes	184 (29.8)	173 (31.6)	11 (15.5)	0.38 [0.18-0.79]	
**Buffalo or cow ownership**					0.97
No	565 (91.4)	500 (91.4)	65 (91.5)	1 [1-1]	
Yes	53 (8.6)	47 (8.6)	6 (8.5)	0.9 [0.31-2.62]	
Household water sources					
**Household collects water at shallow well or stream in dry season**					0.1
No	153 (24.8)	141 (25.8)	12 (16.9)	1 [1-1]	
Yes	465 (75.2)	406 (74.2)	59 (83.1)	1.85 [0.88-3.91]	
**Household collects water at shallow well or stream in rainy season**					0.19
No	226 (36.6)	205 (37.5)	21 (29.6)	1 [1-1]	
Yes	392 (63.4)	342 (62.5)	50 (70.4)	1.55 [0.82-2.92]	
Household agriculture					
**Household grows cassava crop**					0.03
No	306 (50)	263 (48.4)	43 (62.3)	1 [1-1]	
Yes	306 (50)	280 (51.6)	26 (37.7)	0.56 [0.31-1.00]	
**Household grows soybean crop**					<0.0001
No	446 (72.9)	383 (70.5)	63 (91.3)	1 [1-1]	
Yes	166 (27.1)	160 (29.5)	6 (8.7)	0.21 [0.085-0.53]	
**Household grows peanut crop**					0.09
No	601 (98.2)	535 (98.5)	66 (95.7)	1 [1-1]	
Yes	11 (1.8)	8 (1.5)	3 (4.3)	3.06 [0.53-17.6]	
**Household grows cashew crop**					0.81
No	170 (27.8)	150 (27.6)	20 (29)	1 [1-1]	
Yes	442 (72.2)	393 (72.4)	49 (71)	0.95 [0.50-1.82]	
**Household growns green bean crop**					0.75
No	600 (98)	532 (98)	68 (98.6)	1 [1-1]	
Yes	12 (2)	11 (2)	1 (1.4)	0.64 [0.062-6.71]	

**Household grows rubber crop**						0.47
No	608 (99.3)	539 (99.3)	69 (100)	1	[1-1]	
Yes	4 (0.7)	4 (0.7)	0 (0)	1	[1-1]	
**Household grows sesame crop**						0.1
No	549 (89.7)	491 (90.4)	58 (84.1)	1	[1-1]	
Yes	63 (10.3)	52 (9.6)	11 (15.9)	1.93	[0.82-4.54]	
**Household member stayed overnight at cashew or rubber plantation**						0.09
No	240 (38.8)	219 (40)	21 (29.6)	1	[1-1]	
Yes	378 (61.2)	328 (60)	50 (70.4)	1.65	[0.89-3.05]	
**Household hosted hired workers in past year**						0.37
No	595 (96.3)	528 (96.5)	67 (94.4)	1	[1-1]	
Yes	23 (3.7)	19 (3.5)	4 (5.6)	1.67	[0.43-6.40]	

Table S3: Unadjusted odds ratios for association between individual-level variables and odds of *Plasmodium* infection (children)

Variable	n (%)			OR [95% CI]	p-value
	Total (%)	*Plasmodium* -	*Plasmodium* +		
**Village**					0.04
Chamkar San	172 (42.6)	165 (44.4)	7 (21.9)	1 [1-1]	
Phi	143 (35.4)	126 (33.9)	17 (53.1)	4.14 [1.23-14.0]	
Tun	89 (22)	81 (21.8)	8 (25)	2.64 [0.70-9.98]	
**Age**					0.0006
0-3	115 (28.5)	113 (30.4)	2 (6.2)	1 [1-1]	
4-7	146 (36.1)	136 (36.6)	10 (31.2)	3.63 [0.69-19.1]	
8-11	143 (35.4)	123 (33.1)	20 (62.5)	11.4 [2.23-58.5]	
**Sex**					0.5
Male	207 (51.2)	192 (51.6)	15 (46.9)	1 [1-1]	
Female	197 (48.8)	180 (48.4)	17 (53.1)	1.34 [0.57-3.15]	
**Ethnicity**					N/A
Ethnic minority	375 (94.9)	343 (94.5)	32 (100)	1 [1-1]	
Khmer	20 (5.1)	20 (5.5)	0 (0)	1 [1-1]	
**Frequency of village sleeping in dry season**					0.14
Every night	104 (26)	101 (27.4)	3 (9.4)	1 [1-1]	
At least weekly	109 (27.3)	101 (27.4)	8 (25)	2.81 [0.60-13.3]	
Monthly or less	89 (22.2)	79 (21.5)	10 (31.2)	4.62 [0.99-21.5]	
Never	98 (24.5)	87 (23.6)	11 (34.4)	4.64 [1.02-21.2]	
**Frequency of village sleeping in rainy season**					0.07
Every night	100 (25)	98 (26.6)	2 (6.2)	1 [1-1]	
At least weekly	84 (21)	77 (20.9)	7 (21.9)	4.96 [0.83-29.6]	
Monthly or less	98 (24.5)	86 (23.4)	12 (37.5)	7.36 [1.34-40.3]	
Never	118 (29.5)	107 (29.1)	11 (34.4)	5.4 [0.99-29.5]	
**Frequency of farm sleeping in dry season**					0.12
Every night	100 (25)	90 (24.5)	10 (31.2)	1 [1-1]	
At least weekly	133 (33.2)	119 (32.3)	14 (43.8)	1.1 [0.36-3.38]	
Monthly or less	55 (13.8)	50 (13.6)	5 (15.6)	0.89 [0.21-3.77]	
Never	112 (28)	109 (29.6)	3 (9.4)	0.23 [0.050-1.07]	

**Frequency of farm sleeping in rainy season**						0.08
Every night	118 (29.5)	107 (29.1)	11 (34.4)	1	[1-1]	
At least weekly	147 (36.8)	131 (35.6)	16 (50)	1.2	[0.42-3.42]	
Monthly or less	37 (9.2)	34 (9.2)	3 (9.4)	0.84	[0.16-4.41]	
Never	98 (24.5)	96 (26.1)	2 (6.2)	0.19	[0.033-1.03]	
**Bed net use night before survey**						0.38
No BN	6 (1.5)	4 (1.1)	2 (6.2)	6.99	[0.50- 98.39]	
ITN/LLIN	238 (60.3)	219 (60.3)	19 (59.4)	1	[1-1]	
Market net	151 (38.2)	140 (38.6)	11 (34.4)	0.97	[0.36-2.58]	
**Frequency of outdoor evening child play**						0.82
Every night	306 (75.7)	281 (75.5)	25 (78.1)	1	[1-1]	
At least weekly	62 (15.3)	57 (15.3)	5 (15.6)	1.01	[0.29-3.45]	
Monthly or less	36 (8.9)	34 (9.1)	2 (6.2)	0.58	[0.092-3.65]	
**Frequency of indoor evening play outside of bed net**						0.76
Every night	293 (72.5)	269 (72.3)	24 (75)	1	[1-1]	
At least weekly	60 (14.9)	55 (14.8)	5 (15.6)	0.94	[0.26-3.39]	
Monthly or less	51 (12.6)	48 (12.9)	3 (9.4)	0.56	[0.11-2.81]	
**Sleeping time**						0.25
Before 8pm	211 (52.2)	197 (53)	14 (43.8)	1	[1-1]	
8pm or later	193 (47.8)	175 (47)	18 (56.2)	1.71	[0.67-4.36]	

Table S4: Unadjusted odds ratios for association between household-level variables and odds of *Plasmodium* infection (children)

Variable	n (%)			OR	[95% CI]	p-value
	Total (%)	*Plasmodium* -	*Plasmodium* +			
			Household characteristics			
**Household size**						0.84
0-3	29 (7.4)	28 (7.8)	1 (3.1)	1	[1-1]	
4-5	116 (29.7)	105 (29.2)	11 (34.4)	3.28	[0.31-35.0]	
6-7	137 (35)	125 (34.8)	12 (37.5)	3.06	[0.29-32.5]	
8-9	62 (15.9)	58 (16.2)	4 (12.5)	2.1	[0.16-27.6]	
10 or more	47 (12)	43 (12)	4 (12.5)	3.05	[0.22-42.2]	
**Socioeconomic status**						
First (lowest)	89 (22.4)	80 (21.8)	9 (29)	1	[1-1]	
Second	97 (24.4)	92 (25.1)	5 (16.1)	0.43	[0.098-1.93]	
Third	153 (38.4)	137 (37.3)	16 (51.6)	1.18	[0.35-3.91]	
Fouth (highest)	59 (14.8)	58 (15.8)	1 (3.2)	0.13	[0.012-1.46]	
			House construction characteristics			
**Number of steps from ground to house**						0.99
On the ground	78 (19.6)	72 (19.6)	6 (19.4)	1	[1-1]	
1-5 steps	169 (42.5)	155 (42.2)	14 (45.2)	1.27	[0.32-5.03]	
6-9 steps	98 (24.6)	91 (24.8)	7 (22.6)	1.04	[0.22-4.95]	
10 or more steps	53 (13.3)	49 (13.4)	4 (12.9)	1.06	[0.18-6.30]	
**House main roof material**						0.28
Plant/plastic	63 (15.8)	56 (15.3)	7 (22.6)	1	[1-1]	
Tin/Tile	335 (84.2)	311 (84.7)	24 (77.4)	0.69	[0.20-2.40]	
**House main wall material**						0.07
Plant/plastic	123 (30.9)	109 (29.7)	14 (45.2)	1	[1-1]	
Wood/tin	275 (69.1)	258 (70.3)	17 (54.8)	0.54	[0.20-1.43]	
**House main floor material**						0.09
Bamboo/earth	81 (20.4)	71 (19.3)	10 (32.3)	1	[1-1]	
Wood/concrete	317 (79.6)	296 (80.7)	21 (67.7)	0.52	[0.17-1.54]	
**House has open walls**						0.23
No	361 (90.7)	331 (90.2)	30 (96.8)	1	[1-1]	
Yes	37 (9.3)	36 (9.8)	1 (3.2)	0.29	[0.029-2.98]	

			Household assets			
**Total number of village houses**						0.92
0	79 (19.8)	72 (19.6)	7 (22.6)	1	[1-1]	
1	315 (79.1)	291 (79.3)	24 (77.4)	0.84	[0.25-2.83]	
2 to 3	4 (1.0)	4 (1.0)	0 (0)	1	[1-1]	
**Total number of farm houses**						0.17
0	95 (23.9)	93 (25.3)	2 (6.5)	1	[1-1]	
1	262 (65.8)	236 (64.3)	26 (83.9)	5.41	[1.07-27.4]	
2	35 (8.8)	32 (8.7)	3 (9.7)	5.02	[0.59-42.3]	
3	2 (0.5)	2 (0.5)	0 (0)	1	[1-1]	
4	4 (1)	4 (1.1)	0 (0)	1	[1-1]	
**Total number of field houses**						0.15
0	375 (94.2)	344 (93.7)	31 (100)	1	[1-1]	
1	23 (5.8)	23 (6.3)	0 (0)	1	[1-1]	
**Car ownership**						0.33
No	387 (97.2)	356 (97)	31 (100)	1	[1-1]	
Yes	11 (2.8)	11 (3)	0 (0)	1	[1-1]	
**Motorbike ownership**						0.4
No	68 (17.1)	61 (16.6)	7 (22.6)	1	[1-1]	
Yes	330 (82.9)	306 (83.4)	24 (77.4)	0.62	[0.18-2.16]	
**Bicycle ownership**						0.83
No	315 (79.1)	290 (79)	25 (80.6)	1	[1-1]	
Yes	83 (20.9)	77 (21)	6 (19.4)	0.84	[0.24-2.93]	
**TV ownership**						0.59
No	347 (87.2)	319 (86.9)	28 (90.3)	1	[1-1]	
Yes	51 (12.8)	48 (13.1)	3 (9.7)	0.75	[0.16-3.52]	
				398		
**DVD/MP3 player ownership**						
No	228 (57.3)	208 (56.7)	20 (64.5)	1	[1-1]	
Yes	170 (42.7)	159 (43.3)	11 (35.5)	0.67	[0.24-1.87]	
**Satellite dish ownership**						0.44
No	391 (98.2)	360 (98.1)	31 (100)	1	[1-1]	
Yes	7 (1.8)	7 (1.9)	0 (0)	1	[1-1]	

**Generator ownership**						0.12
No	351 (88.2)	321 (87.5)	30 (96.8)	1	[1-1]	
Yes	47 (11.8)	46 (12.5)	1 (3.2)	0.21	[0.022-2.06]	
**Solar panel ownership**						0.28
No	217 (54.5)	203 (55.3)	14 (45.2)	1	[1-1]	
Yes	181 (45.5)	164 (44.7)	17 (54.8)	1.7	[0.62-4.64]	
**Home battery ownership**						0.52
No	96 (24.1)	90 (24.5)	6 (19.4)	1	[1-1]	
Yes	302 (75.9)	277 (75.5)	25 (80.6)	1.43	[0.41-4.92]	
**Farm machine ownership**						0.3
No	289 (72.6)	264 (71.9)	25 (80.6)	1	[1-1]	
Yes	109 (27.4)	103 (28.1)	6 (19.4)	0.46	[0.13-1.66]	
**Buffalo or cow ownership**						0.71
No	378 (95)	349 (95.1)	29 (93.5)	1	[1-1]	
Yes	20 (5)	18 (4.9)	2 (6.5)	1.45	[0.19-11.1]	
		Household water sources				
**Household collects water at shallow well or stream in dry season**						0.01
No	119 (29.9)	116 (31.6)	3 (9.7)	1	[1-1]	
Yes	279 (70.1)	251 (68.4)	28 (90.3)	4.9	[1.20-19.9]	
**Household collects water at shallow well or stream in rainy season**						0.03
No	149 (37.4)	143 (39)	6 (19.4)	1	[1-1]	
Yes	249 (62.6)	224 (61)	25 (80.6)	2.65	[0.89-7.92]	
		Household agriculture				
**Household grows cassava crop**						0.38
No	174 (44.3)	163 (44.9)	11 (36.7)	1	[1-1]	
Yes	219 (55.7)	200 (55.1)	19 (63.3)	1.61	[0.55-4.77]	
**Household grows soybean crop**						<0.0001
No	250 (63.6)	221 (60.9)	29 (96.7)	1	[1-1]	
Yes	143 (36.4)	142 (39.1)	1 (3.3)	0.04	[0.0049-0.37]	

**Household grows peanut crop**						0.1
No	384 (97.7)	356 (98.1)	28 (93.3)	1	[1-1]	
Yes	9 (2.3)	7 (1.9)	2 (6.7)	5.05	[0.33-77.6]	
**Household grows cashew crop**						0.53
No	124 (31.6)	113 (31.1)	11 (36.7)	1	[1-1]	
Yes	269 (68.4)	250 (68.9)	19 (63.3)	0.87	[0.29-2.61]	
**Household growns green bean crop**						0.38
No	384 (97.7)	354 (97.5)	30 (100)	1	[1-1]	
Yes	9 (2.3)	9 (2.5)	0 (0)	1	[1-1]	
**Household grows rubber crop**						0.62
No	390 (99.2)	360 (99.2)	30 (100)	1	[1-1]	
Yes	3 (0.8)	3 (0.8)	0 (0)	1	[1-1]	
**Household grows other crop**						0.78
No	323 (90)	301 (89.9)	22 (91.7)	1	[1-1]	
Yes	36 (10)	34 (10.1)	2 (8.3)	0.87	[0.14-5.28]	
**Household grows sesame crop**						0.8
No	362 (92.1)	334 (92)	28 (93.3)	1	[1-1]	
Yes	31 (7.9)	29 (8)	2 (6.7)	0.9	[0.13-6.07]	
**Household member stayed overnight at cashew or rubber plantation**						0.04
No	147 (36.9)	141 (38.4)	6 (19.4)	1	[1-1]	
Yes	251 (63.1)	226 (61.6)	25 (80.6)	2.77	[0.91-8.45]	
**Household hosted hired workers in past year**						0.38
No	389 (97.7)	358 (97.5)	31 (100)	1	[1-1]	
Yes	9 (2.3)	9 (2.5)	0 (0)	1	[1-1]	
